# Implementation of a Pooled Surveillance Testing Program for Asymptomatic SARS-CoV-2 Infections on a College Campus — Duke University, Durham, North Carolina, August 2–October 11, 2020

**DOI:** 10.15585/mmwr.mm6946e1

**Published:** 2020-11-20

**Authors:** Thomas N. Denny, Laura Andrews, Mattia Bonsignori, Kyle Cavanaugh, Michael B. Datto, Anastasia Deckard, C. Todd DeMarco, Nicole DeNaeyer, Carol A. Epling, Thaddeus Gurley, Steven B. Haase, Chloe Hallberg, John Harer, Charles L. Kneifel, Mark J. Lee, Raul Louzao, M. Anthony Moody, Zack Moore, Christopher R. Polage, Jamie Puglin, P. Hunter Spotts, John A. Vaughn, Cameron R. Wolfe

**Affiliations:** ^1^Duke Human Vaccine Institute, Duke University School of Medicine, Durham, North Carolina; ^2^Student Affairs, Duke University, Durham, North Carolina; ^3^Department of Medicine, Duke University School of Medicine, Durham, North Carolina; ^4^Department of Family Medicine, Duke University School of Medicine, Durham, North Carolina; ^5^Department of Pathology, Duke University of Medicine, Durham, North Carolina; ^6^Office of Information Technology, Duke University, Durham, North Carolina; ^7^Emergency Management and Emergency Services, Duke University, Durham, North Carolina; ^8^Department of Mathematics, Duke University, Durham, North Carolina; ^9^Department of Pediatrics, Duke University School of Medicine, Durham, North Carolina; ^10^North Carolina Department of Health and Human Services; ^11^Office of Assessment, Duke University, Durham, North Carolina.

*On November 17, 2020, this report was posted online as an *MMWR* Early Release.*

On university campuses and in similar congregate environments, surveillance testing of asymptomatic persons is a critical strategy (*1*,*2*) for preventing transmission of SARS-CoV-2, the virus that causes coronavirus disease 2019 (COVID-19). All students at Duke University, a private research university in Durham, North Carolina, signed the Duke Compact (*3*), agreeing to observe mandatory masking, social distancing, and participation in entry and surveillance testing. The university implemented a five-to-one pooled testing program for SARS-CoV-2 using a quantitative, in-house, laboratory-developed, real-time reverse transcription–polymerase chain reaction (RT-PCR) test (*4*,*5*). Pooling of specimens to enable large-scale testing while minimizing use of reagents was pioneered during the human immunodeficiency virus pandemic (*6*). A similar methodology was adapted for Duke University’s asymptomatic testing program. The baseline SARS-CoV-2 testing plan was to distribute tests geospatially and temporally across on- and off-campus student populations. By September 20, 2020, asymptomatic testing was scaled up to testing targets, which include testing for residential undergraduates twice weekly, off-campus undergraduates one to two times per week, and graduate students approximately once weekly. In addition, in response to newly identified positive test results, testing was focused in locations or within cohorts where data suggested an increased risk for transmission. Scale-up over 4 weeks entailed redeploying staff members to prepare 15 campus testing sites for specimen collection, developing information management tools, and repurposing laboratory automation to establish an asymptomatic surveillance system. During August 2–October 11, 68,913 specimens from 10,265 graduate and undergraduate students were tested. Eighty-four specimens were positive for SARS-CoV-2, and 51% were among persons with no symptoms. Testing as a result of contact tracing identified 27.4% of infections. A combination of risk-reduction strategies and frequent surveillance testing likely contributed to a prolonged period of low transmission on campus. These findings highlight the importance of combined testing and contact tracing strategies beyond symptomatic testing, in association with other preventive measures. Pooled testing balances resource availability with supply-chain disruptions, high throughput with high sensitivity, and rapid turnaround with an acceptable workload.

Duke’s SARS-CoV-2 surveillance program commenced when the campus reopened for fall 2020 classes. As advised by the Atlantic Coast Conference Medical Advisory Group, a total of 781 student-athletes and student athletic assistants have been participating in a separate surveillance program, in which teams are categorized as high-, medium-, or low-risk. Results described here focus on testing of students who are not student-athletes. The pooled testing program was aimed at students, but was also available to faculty and staff members. Not included are the results of specimens tested from 8,012 faculty and staff members (including pooled tests of specimens from asymptomatic persons and individual testing of specimens from symptomatic persons) by mid-September.

Students self-quarantined at home for 14 days before arriving at the reopened campus in scheduled windows during August 11–15. The surveillance program includes entry testing for all incoming students, surveillance of asymptomatic persons using pooled testing, and individual testing for symptomatic persons. Upon arrival, all students underwent entry SARS-CoV-2 screening that included collection of nasopharyngeal swabs that were tested using standard protocols in a CAP/CLIA-certified* laboratory; students were sequestered in prearranged housing (dormitories or off-campus housing) pending results (*7*). The students who were already in residence on campus or in the local community did not participate in entry testing. Mitigation strategies included converting all dormitory rooms to single-occupancy, modifying classrooms and common areas to accommodate social distancing, and distributing packaged meals. All students signed the Duke Compact (*3*), agreeing to observe mandatory masking, social distancing, and participation in entry and surveillance testing. Students who missed scheduled surveillance tests lost access to campus facilities and services. Compliance for testing among students on the date requested was approximately 95% (*8*). In addition, contact tracing was performed for all positive cases. Exposed contacts were quarantined for 14 days, and students, whether asymptomatic or symptomatic, submitted specimens for testing upon initiating quarantine and again if they became symptomatic during quarantine.

Students also installed the custom-built SymMon (symptom monitoring) smartphone app,^†^ which administers a daily symptom survey (*7*). The app facilitates testing for symptomatic users and for asymptomatic persons undergoing pooled testing. The app’s barcode scanner enables linking of specimens to persons and creation of labels for electronic health record system orders. In addition to students, all faculty and staff members were required to complete the same SymMon symptom survey before arrival on each day they entered the campus.

Duke’s SARS-CoV-2 surveillance program is ongoing. Testing of nasal swabs collected from symptomatic persons is conducted in a CAP/CLIA–certified laboratory using a platform approved under the Food and Drug Administration (FDA) Emergency Use Authorization (EUA). Testing sites for asymptomatic persons receive prelabeled tubes, swabs, and specimen bags. Supervised self-collected nasal swabs are obtained,^§^ and unique barcodes are scanned using the SymMon app to record date and time and establish the link between person and specimen. Specimens are placed in secondary containers and driven to the processing laboratory. Testing of asymptomatic persons reached full capacity on September 20; since then, residential undergraduates are tested twice weekly, off-campus undergraduates one to two times per week, and graduate students approximately once weekly. At full capacity during weeks 6–9, an average of 11,390 samples were pooled per week (2,278 samples per day, 5 days per week).

Laboratory automation was rapidly repurposed to provide a high-throughput, rapid platform for pooling specimens for RT-PCR testing. An automated five-to-one pooling run transfers 120 primary samples into 24 2-mL tubes in 13 minutes, 9 seconds (33 seconds per pool). After pooling, specimens are held at 39.2°F (4°C) pending final disposition. Pooled samples are tested using an automated QIAsymphony (Qiagen LLC) laboratory-developed two-step RT-PCR and the World Health Organization E_Sarbeco primer-probe set (Charité/Berlin).^¶^ For pooled assays, viral load calibration standards are run on each plate, and positive pool viral loads are extrapolated from the calibration curve (Table 1). Clinical viral loads are reported from a similar calibration process.

**TABLE 1:** Validation data* for the SARS-CoV-2 quantitative viral load assay indicating 100% target detection at 62 copies/mL and 74% at 15 copies/mL — Duke University, Durham, North Carolina, August–October 2020

Sample ID^†^	Target viral load (RNA copies/mL)	% Detection (95% CI)	
		Both replicates detected	Single replicate detected
Validation panel A	5,000,000	100 (94.9–NE)	100 (94.9–NE)
Validation panel B	500,000	100 (94.9–NE)	100 (94.9–NE)
Validation panel C	50,000	100 (94.9–NE)	100 (94.9–NE)
Validation panel D	5,000	100 (94.9–NE)	100 (94.9–NE)
Validation panel E	500	100 (94.9–NE)	100 (94.9–NE)
Validation panel F	250	100 (94.9–NE)	100 (94.9–NE)
Validation panel G	125	99 (92.3–99.9)	100 (94.9–NE)
Validation panel H	62	83 (72.0–91.0)	100 (94.9–NE)
Validation panel I	31	56 (43.3–68.6)	94 (86.0–98.4)
Validation panel J	15	27 (17.2–39.1)	74 (62.0–84.0)

**Abbreviations:** CI = confidence interval; NE = not able to estimate.

* Validation panels were tested 70 times to determine limit of detection with 95% CIs.

^†^ Genomic viral RNA was used to establish the validation panels.

Positive pools are flagged for follow-up by deconvolution (individual testing of specimens in positive pools). For each pool, the five component specimens are retrieved, aliquoted, and labeled with unique barcodes. SymMon data are used to generate clinical orders, and specimens are tested in the CLIA-certified Duke Clinical Microbiology Laboratory using standard protocols. Results are entered into electronic health records and reported to the University’s Student Health. Clinical assays (Xpert-Xpress SARS-COV-2 [Cepheid], Abbott Alinity mSARS-COv-2 [Abbott Diagnostics] or Roche cobas SARS-COV-2 [Roche Diagnostics]) were authorized for emergency use by the FDA. The two-stage testing strategy described here was designed so that the first stage used a sensitive test in a low-prevalence population, and the second stage used EUA clinical tests in the identified subset of samples where the pretest probability was higher. Additional details regarding clinical assays, sample pooling, and testing are available online.**

During August 2–October 11, a total of 10,265 undergraduate and graduate students, representing all students residing on campus or in the Durham community, but excluding athletes (781) and students attending class remotely outside of Durham (4,452), participated in pooled testing. Overall, 68,913 tests were performed for students, including 8,873 entry tests (1,392 students were already in residence on campus or in the Durham community), 59,476 pooled tests, 379 contact-traced tests, and 185 tests for symptomatic students (Table 2).

**TABLE 2:** Number of tests* positive for SARS-CoV-2 among students, by test category — Duke University, Durham, North Carolina, August 2–October 11, 2020

Test category	No. of tests performed	No. of positive tests	No. (%) of persons† asymptomatic at testing
Entry testing	8,873	17	9 (53)
Pooled testing^§^	59,476	29	29 (100)
Contact tracing^¶^	379	23	5 (22)
Symptom monitoring^¶^	185	15	0 (0)
**Total**	**68,913**	**84**	**43 (51)**

* Testing was performed on specimens from a total population of 10,265 undergraduate and graduate students residing on Duke University campus or in the surrounding Durham community.

^†^ Who received positive test results.

^§^ Total number of positive pools = 158, which upon deconvolution yielded 29 individual positive specimens among students.

^¶^ Because numbers for total tests in contact tracing and symptom monitoring were encoded together, classifications of tests as resulting from contact tracing or symptom monitoring in this table represent an estimate.

Duke’s comprehensive strategy includes multiple categories of tests to identify COVID-19 infections (Table 2). During August 2–October 11, a total of 84 cases among students were identified. Across testing categories, 17 cases (20.2%) were detected by entry testing (nine asymptomatic and eight symptomatic), 29 cases (34.5%) by pooled testing (all asymptomatic), 23 cases (27.4%) by contact tracing (five asymptomatic and 18 symptomatic at time of testing), and 15 (17.9%) by symptom monitoring. Overall, among 84 total students who received positive test results, 43 (51%) did not report symptoms at the time of testing (Table 2).

Contact tracing was activated for each case detected. Among 379 students quarantined as a result of contact tracing, 23 (6.1%) received positive test results while in quarantine. Thus, the combined number of cases in asymptomatic students identified by testing (entry and pooled) and cases in all students identified by contact tracing accounted for 61 (73%) of the 84 COVID-19 cases that might not have been detected as rapidly or completely through symptomatic testing alone. Because of high testing frequency, an accurate weekly per-capita infection incidence was calculated, averaging 0.08% during the measurement period. Pooled testing for asymptomatic students comprises two steps: pooled screening and deconvolution. The pooled screening resulted in 158 positive pools that, upon deconvolution, identified 29 (18.4%) confirmed cases.

 Estimated viral load was reported for pooled tests and clinical deconvolution tests. Specimens that tested positive upon deconvolution indicated good concordance with viral load estimates for positive pools (Figure). Viral load estimates for multiple asymptomatic students reached levels >10,000,000 copies/mL (geometric mean = 2,590 copies/mL [range = 3–32,360,000 copies/mL]). For pooled testing, the time between sampling, return of a positive pool, subsequent deconvolution, and return of clinical results was 18–30 hours. In addition, pooled testing permitted a nearly 80% savings in use of reagents and laboratory resources compared with testing each individual specimen.

**FIGURE:**
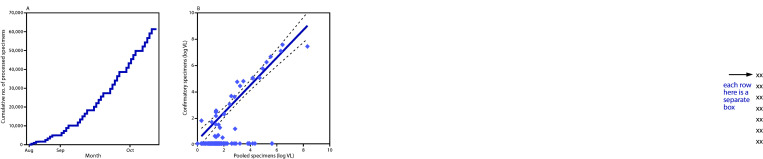
Cumulative number of nasal swab specimens processed for pooled SARS-CoV-2 real-time reverse transcription–polymerase chain reaction testing, August 18–October 11, 2020 (A) and viral load estimates for pooled (n = 158) and confirmatory specimens (n = 30), August–October 2020 (B)* — Duke University, Durham, North Carolina **Abbreviation:** VL = viral load. * In addition to data for students, plot includes data for one faculty member with a positive test result.

## Discussion

For the fall 2020 semester at Duke University, COVID-19 mitigation strategies included mandatory mask wearing, social distancing, emphasis of hand hygiene, daily symptom self-monitoring/reporting, and a multipronged testing strategy that comprised entry testing of all students, frequent testing of pooled student specimens, contact tracing with quarantine, and testing for symptomatic and exposed students. The cross-sectional strategy for collecting surveillance/pooled testing specimens involved distributing tests weekly across off- and on-campus student populations. In addition, the frequency of surveillance/pooled testing enabled real-time adaptive sampling, wherein additional individual specimens were focused either geospatially or within identified cohorts of the persons with positive test results. Case identification activated contact tracing for quarantine and testing for exposed asymptomatic contacts. This plan allowed campus to remain open for 10 weeks of classes without substantial outbreaks among residential or off-campus populations. Importantly, no evidence from contact tracing linked transmission with in-person classes.

Multiple universities began fall 2020 classes using only symptomatic testing. Among colleges with in-person classes and approximately 5,000 undergraduates, only 6% routinely tested all of their students in the fall semester.^††^ The finding that 51% of SARS-CoV-2 infections in this analysis were asymptomatic suggests that a substantial proportion of infections would be missed with only symptomatic testing. Entry and pooled testing of asymptomatic students combined with contact tracing allowed identification and isolation of nearly three quarters of students with diagnosed infections. Importantly, despite constrained testing resources, pooled surveillance enabled the data-driven deployment of testing to areas or groups potentially at risk for an outbreak before substantial spread. Frequent testing in addition to asymptomatic entry testing, facilitated isolation of infected students before transmission could occur, keeping baseline incidence low; average weekly per-capita incidence among students was estimated to be 0.08% (*8*). By comparison, during October 12–18, weekly per-capita positivity for Durham County was 0.1% (*9*). Several asymptomatic students had high viral loads, suggesting substantial potential for transmission (*1*). These findings highlight the importance of combined testing and tracing strategies beyond symptomatic testing.

Recently, a COVID-19 cluster involving multiple students was identified in off-campus housing. Pooled testing identified the asymptomatic index patient. After contact tracing identified students with potential exposure, eight students linked to the index patient received positive test results. Pooled testing and contact tracing rapidly isolated the cluster, preventing further transmission. In addition, rapid identification of cases among contacts in off-campus locations might have prevented community outbreaks.

The high sensitivity of RT-PCR testing could support use of larger pools or more complex two-stage testing strategies than those used in this study. However, deconvolution would also increase turnaround time, reducing capacity for rapid identification and isolation of infections. Using five-to-one pooling balances resource availability with supply-chain disruptions, high throughput with high sensitivity, and rapid turnaround with an acceptable workload for the laboratory conducting confirmatory testing. Further, surveillance testing at this scale in a relatively low-prevalence population will identify more false positives than true positives; thus, the current two-stage approach and pooling size allows rapid identification and confirmation of asymptomatic cases for contact tracing.

The findings of this report are subject to at least four limitations. First, the determination of whether students were asymptomatic or symptomatic at the time of testing relied on self-reporting of symptoms, which was unlikely to be fully accurate. Second, some reported positive cases might have included students who were not residing on campus or within the Durham, North Carolina, community at the time of the report. Third, positive pools were deconvoluted in a CLIA-certified clinical laboratory using multiple EUA-certified platforms with different metrics and thresholds for determining positives. Finally, the impact of Duke’s testing program was assessed within the context of an incidence rate specific to the local Durham community and in the context of multiple strategies for mitigations on campus. The precise findings were likely influenced by multiple factors, such as maintaining students in single rooms on campus and by the level of adherence to campus policies on face coverings, social distancing, and symptom monitoring by Duke’s student populations.

Before fall 2020, many universities made decisions based on epidemiologic models with scant data for estimating critical parameters (*2*,*10*). Among the Duke student body and faculty and staff members, weekly or more frequent mandatory testing led to low infection rates when combined with preventive mitigation strategies such as frequent handwashing, masking, and social distancing. In addition to limiting transmission on campus and within the local community, Duke’s comprehensive COVID-19 mitigation will provide critical data to inform parameters in epidemiologic models and support data-driven approaches on college campuses and in other settings.

**Summary**

**What is already known about this topic?**

SARS-CoV-2 can rapidly spread through university settings. Pooling specimens can enable large-scale testing while minimizing needed resources.

**What is added by this report?**

In fall 2020, Duke University’s COVID-19 prevention strategy included risk reduction behaviors, frequent testing using pooled SARS-CoV-2 polymerase chain reaction testing, and contact tracing. Among 10,265 students who received testing 68,913 times, 84 had positive results. One half of infections were asymptomatic, and some had high viral loads.

**What are the implications for public health practice?**

SARS-CoV-2 transmission was limited in this congregate setting by integration of prevention strategies that included identification of asymptomatic infections through frequent testing. Pooled testing reduced the need for resources while allowing high throughput with high sensitivity and rapid turnaround of results.

## Footnotes

* College of American Pathologists (https://www.cap.org/); Clinical Laboratory Improvement Amendments (https://www.cdc.gov/clia/about.html).

† SymMon app developed by Mike Revoir, Matt Gardner, Shellene Walker, and Scott Barkie, Office of Information Technology and Institute for Health Innovation, Duke University.

§ https://www.fda.gov/news-events/press-announcements/coronavirus-covid-19-update-fda-authorizes-first-test-patient-home-sample-collection.

¶ https://www.who.int/docs/default-source/coronaviruse/protocol-v2-1.pdf.

** https://iqa.center.duke.edu/sites/iqa.center.duke.edu/files/Online%20Methods%20Supplement%20%2800000002%29.docx.

†† https://www.npr.org/2020/10/06/919159473/even-in-covid-hot-spots-many-colleges-arent-aggressively-testing-students.
